# Hospital mortality and length of stay associated with *Enterobacterales* positive blood cultures: a multicenter analysis

**DOI:** 10.1128/spectrum.00402-24

**Published:** 2024-07-02

**Authors:** Lauren Cooper, Kalvin Yu, Kayla Van Benten, Anuprita Patkar, Gang Ye, Sara Gregory, ChinEn Ai, Vikas Gupta

**Affiliations:** 1 Becton, Dickinson and Company, Diagnostic Solutions, Sparks, Maryland, USA; 2 Becton, Dickinson and Company, Franklin Lakes, New Jersey, USA; Emory University School of Medicine, Atlanta, Georgia, USA

**Keywords:** bloodstream infections, carbapenem, antibiotic resistance, *Enterobacterales*, empiric therapy

## Abstract

Delayed time to antimicrobial susceptibility results can impact patients’ outcomes. Our study evaluated the impact of susceptibility turnaround time (TAT) and inadequate empiric antibacterial therapy (IET) in patients with bloodstream infections (BSI) caused by *Enterobacterales* (ENT) species on in-hospital mortality and length of stay (LOS). This retrospective, multicenter investigation which included 29,570 blood ENT-positive admissions across 161 US healthcare facilities evaluated the association between antimicrobial susceptibility testing (AST) TAT, carbapenem susceptibility, and empiric therapy on post-BSI in-hospital mortality and LOS following an ENT BSI event in adult patients. After adjusting for outcomes covariates, post-BSI in-hospital mortality was significantly higher for patients in the IET vs adequate empiric therapy (AET) group [odds ratio (OR): 1.61 (95% CI: 1.32, 1.98); *P* < 0.0001], and when AST TAT was >63 h [OR:1.48 (95% CI: 1.16, 1.90); *P* = 0.0017]. Patients with carbapenem non-susceptible (carb-NS) ENT BSI had significantly higher LOS (16.6 days, 95% CI: 15.6, 17.8) compared to carbapenem susceptible (carb-S, 12.2 days, 95% CI: 11.8, 12.6), (*P* < 0.0001). Extended AST TAT was significantly associated with longer LOS for TAT of 57–65 h and >65 h (*P* = 0.005 and *P*< 0.0001, respectively) compared to TAT ≤42 h (reference). Inadequate empiric therapy (IET), carb-NS, and delayed AST TAT are significantly associated with adverse hospital outcomes in ENT BSI. Workflows that accelerate AST TAT for ENT BSIs and facilitate timely and adequate therapy may reduce post-BSI in-hospital mortality rate and LOS.

**IMPORTANCE:**

For patients diagnosed with bloodstream infections (BSI) caused by *Enterobacterales* (ENT), delayed time to antimicrobial susceptibility (AST) results can significantly impact in-hospital mortality and hospital length of stay. However, this relationship between time elapsed from blood culture collection to AST results has only been assessed, to date, in a limited number of publications. Our study focuses on this important gap using retrospective data from 29,570 blood ENT-positive admissions across 161 healthcare facilities in the US as we believe that a thorough understanding of the dynamic between AST turnaround time, adequacy of empiric therapy, post-BSI event mortality, and hospital length of stay will help guide effective clinical management and optimize outcomes of patients with ENT infections.

## INTRODUCTION

Bloodstream infections (BSI) caused by *Enterobacterales* (ENT) pathogens pose an important clinical challenge, especially when isolates express resistance to antimicrobial agents (1, 2). In 2019, BSI infections were the second leading cause of global death associated with, or attributable to, antibiotic resistance (3). Carbapenem-resistant ENT (CRE) infections, due to their high rates of morbidity and mortality, have also been categorized by the Centers for Disease Control and Prevention (CDC) as an urgent threat to human health (4). In addition, CRE has been classified by the World Health Organization (WHO) as critical priority pathogens requiring new antibiotic development (5). Whereas prompt administration of antimicrobial therapy is a prime factor in ENT-positive patients’ recovery, empiric antimicrobial regimens are often initiated based on clinician preferences or health system formularies, and usually before antimicrobial susceptibility testing (AST) results are known, potentially leading to inadequate antibiotic selection for patients with antimicrobial-resistant BSI (6–8).

Despite advances in rapid diagnostic techniques, automated susceptibility testing remains the recommended method for guiding antimicrobial therapy in patients with bloodstream infection. In this context, we evaluated the impact of delayed susceptibility results and adequacy of antimicrobial therapy on post-event hospital length of stay and mortality. More specifically, time elapsed from blood culture collection to AST results plays a crucial role in effective clinical management and optimizing outcomes of patients with ENT infections; however, this relationship has only been assessed, to date, in a limited number of publications (8).

## MATERIALS AND METHODS

We conducted a multicenter, retrospective cohort analysis involving 161 healthcare facilities in the BD Insights Research Database (Becton, Dickinson and Company, Franklin Lakes, NJ) to evaluate the impact of adequate and inadequate empirical antibiotic therapy, carbapenem susceptible (carb-S) and carbapenem non-susceptible (carb-NS) ENT, and AST result turnaround time (TAT) on post-BSI event mortality and in-hospital length of stay (LOS) of hospitalized individuals with positive blood cultures.

Eligible participants were ≥18 years old with 1 to 365 days of inpatient stay, had a positive blood culture for ENT with susceptibility results, received ≥24 h of antibacterial therapy, and had a discharge or death record between January 1, 2018 and December 31, 2022. The specific ENT species evaluated included the following: *Escherichia coli*, *Klebsiella pneumoniae*, *Klebsiella oxytoca*, *Klebsiella aerogenes*, *Enterobacter cloacae*, *Serratia marcescens*, *Citrobacter freundii*, *Proteus mirabilis*, *Providencia stuartii,* and *Morganella morganii*. The ENT cohort was stratified by carb-S and carb-NS, respectively, with carb-NS defined as intermediate or resistant susceptibility to meropenem, doripenem, imipenem (excluding *Morganella morganii*, *Proteus mirabilis*, and *Providencia stuartii*), or ertapenem.

Antibacterial therapy was initiated within 48 h prior to blood culture collection until the first AST result that did not cover the pathogen bacteria or to which the blood pathogen bacteria was non-susceptible [either deemed intermediately resistant (I) or resistant (R)] was classified as inadequate empiric therapy (IET). The IET group excluded patients taking multiple antibacterials with at least one active therapy. Adequate empiric therapy (AET) was defined as antibacterial therapy prescribed within 48 h prior to blood culture collection until first AST result that did cover the blood pathogen bacteria and was susceptible. AST TAT was calculated as the date/time of first blood pathogen susceptibility results minus the date/time of blood culture collection. Post-BSI event mortality was evaluated in the subset of patients with available related data. Post-BSI mortality was defined as the death of an in-patient post-blood culture collection, whereas post-BSI event LOS was the length of time spent in the hospital post-ENT-positive blood culture collection. All microbiology results evaluated in this study were those of the hospital or health-system reference laboratory facility where the patients were admitted. The study was approved as involving the use of a limited retrospective data set for an epidemiology study, exempted from consent by the New England Institutional Review Board/WCG Human Subjects Research Committee (Wellesley, MA), and conducted in compliance with Health Insurance Portability and Accountability Act requirements. The study results are reported following the *Strengthening the Reporting of Observational Studies in Epidemiology* (STROBE) guidelines (9).

### Statistical analysis

Descriptive statistics of the observed in-hospital mortality and LOS post-BSI event were evaluated for patients demographic information (age, sex), COVID-19 testing status, carbapenem susceptibility status (carb-S/carb-NS), empiric therapy status (AET/IET), culture turnaround time (categorized into four TAT groups using quartiles as cut-off points), intensive care unit (ICU) status (Yes/No), culture collection setting (admission/hospital-onset period), prior 90-day admission (Yes/No), and concurrent clinical conditions. As per a methodology used in previous studies (10, 11), the six clinical conditions were determined based on maximum laboratory values obtained in the first 5 days of culture collection, as per criteria presented in Table S1. In the exploratory phase of analysis, we examined the bivariate relationship between the study outcomes (post-event in-hospital mortality and LOS) and each primary effect to assess IET, carbapenem susceptibility status, TAT, and other covariates using *t*-tests or analysis of variance (ANOVA) for LOS, and Chi-square tests (or Fisher’s exact tests for small counts) for post-BSI in-hospital mortality. Of note, the TAT quartiles for LOS are those of the full study population, whereas quartiles used for post-event mortality calculations are based on available data reported by healthcare facilities.

The multivariable-adjusted effects of IET, carbapenem susceptibility status, or TAT on post-BSI event LOS were evaluated using the generalized linear mixed models (GLMM), with hospitals as a random effect, to account for within-cluster correlation of data and with gamma distribution and logarithm link function to handle right-skewed LOS data. The random intercept logistic regression model was used to assess the association between post-BSI in-hospital mortality and IET, carbapenem susceptibility status, or TAT, adjusting for the covariates listed in Table 1. The final set of variables/covariates was selected based on the statistical significance of each variable and model goodness-of-fit statistics (smaller Akaike Information Criteria and/or Bayesian Information Criteria). All analyses were conducted using SAS version 9.4 (SAS Institute, Cary, NC, USA).

**TABLE 1:** Distribution of observed post-BSI events LOS and in-hospital mortality with positive *Enterobacterales* blood cultures by patient demographic and clinical characteristics^*a*^

Characteristics	Post-BSI event LOS (days)					Post-BSI event mortality	
	N (%)	Q1	Median	Q3	Mean (SD)	N	Events (%)
Age group							
18–49	4,007 (13.5)	5	7	11	10.52 (13.1)	1,784	90 (5.0)
50–64	7,099 (24.0)	5	7	12	10.50 (11.6)	3,120	214 (6.9)
65–75	8,382 (28.3)	5	7	11	9.63 (9.1)	3,773	255 (6.8)
>75	10,082 (34.1)	5	7	10	9.02 (9.98)	4,129	274 (6.6)
Gender							
Female	14,578 (49.3)	5	7	10	9.27 (9.00)	6,468	351 (5.4)
Male	14,992 (50.7)	5	7	12	10.22 (12.0)	6,338	482 (7.6)
COVID							
Not tested	15,605 (52.8)	5	7	10	9.21 (8.45)	3,207	167 (5.2)
Negative	13,078 (44.2)	5	7	11	10.13 (12.4)	8,951	544 (6.1)
Positive	887 (3.0)	6	9	16	13.69 (15.5)	648	122 (18.8)
Carbapenem susceptibility							
Susceptible	29,155 (98.6)	5	7	11	9.63 (10.2)	12,644	803 (6.4)
Non-susceptible	415 (1.4)	6	10	19	18.30 (27.4)	162	30 (18.5)
Empiric therapy							
IET	4,091 (13.8)	5	8	12	10.67 (12.3)	1,682	189 (11.2)
AET	25,479 (86.2)	5	7	11	9.61 (10.3)	11,124	644 (5.8)
ICU							
No	18,765 (63.4)	5	6	9	7.97 (8.3)	8,341	133 (1.6)
Yes	10,805 (36.5)	6	9	15	12.85 (13.2)	4,465	700 (15.7)
Setting							
Admission	26,489 (89.6)	5	7	10	9.14 (7.9)	11,490	573 (5.0)
Hospital	3,081 (10.4)	5	10	17	15.06 (22.8)	1,316	260 (19.8)
Prior 90-day admission							
No	22,681 (76.7)	5	7	10	9.47 (10.6)	10,338	610 (5.9)
Yes	6,889 (23.3)	5	8	12	10.69 (10.6)	2,468	223 (9.0)
AST TAT* (h) for LOS							
0-Q1 (1st Q, ≤42 h)	7,283 (24.6)	5	7	10	9.35 (8.7)	-	-
Q1-Q2 (median, 43–56 h)	7,470 (25.3)	5	7	10	9.38 (12.6)	-	-
Q2-Q3 (3rd Q, 57–65 h)	7,672 (25.9)	5	7	11	9.75 (9.5)	-	-
Q3 over (> 65 h)	7145 (24.2)	5	8	12	10.55 (11.3)	-	-
AST TAT* (h) for post-BSI mortality							
Q1 (1st Q, <40 h)	-	-	-	-	-	2,860	146 (5.1)
Q1-Q2 (median, 40–48 h)	-	-	-	-	-	3,203	174 (5.4)
Q2-Q3 (3rd Q, 49–63 h)	-	-	-	-	-	3,423	208 (6.1)
Q3 over (4th Q, >63 h)	-	-	-	-	-	3,320	305 (9.2)
Lactic acidosis							
No	24,059 (81.4)	5	7	11	9.56 (10.8)	10,347	498 (4.8)
Yes	5,511 (18.6)	5	8	12	10.60 (9.7)	2,459	335 (13.6)
Renal insufficiency							
No	21,519 (72.8)	5	7	10	9.09 (10.5)	9,438	405 (4.3)
Yes	8,051 (27.2)	6	9	14	11.53 (10.9)	3,368	428 (12.7)
Heart failure							
No	25,961 (87.8)	5	7	11	9.50 (10.2)	11,191	619 (5.5)
Yes	3,609 (12.2)	6	8	13	11.56 (13.1)	1,615	214 (13.3)
Liver dysfunction							
No	11,152 (37.7)	5	6	9	8.71 (11.14)	4,870	147 (3.0)
Yes	18,418 (62.3)	5	8	12	10.39 (10.3)	7,936	686 (8.6)
Cytokine storm							
No	26,719 (90.3)	5	7	11	9.63 (10.7)	11,358	641 (5.6)
Yes	2,851 (9.6)	5	8	13	10.85 (9.99)	1,448	192 (13.3)
Immunocompromised							
No	14,015 (47.4)	5	7	11	10.15 (11.2)	5,968	384 (6.4)
Yes	15,555 (52.6)	5	7	11	9.39 (10.1)	6,838	449 (6.6)
Any of the six comorbidities							
No	4,638 (15.7)	5	6	10	8.88 (9.3)	1,913	55 (2.9)
Yes	24,932 (84.3)	5	7	11	9.91 (10.9)	10,893	778 (7.1)

^*a*^ S. susceptible; NS, non-susceptible; IET, inadequate empiric therapy; AST, antimicrobial susceptibility testing; TAT, turnaround time; BSI, bloodstream infection; AET, adequate empiric therapy; LOS, length of stay; Q, quartile. *Quartiles of TAT in the table are those of the full study population (N = 29,570) and may differ from those used for post-event mortality calculations, which are based on available data reported by healthcare facilities.

## RESULTS

In our study, male and female participants were similarly represented (50.7% vs 49.3%, respectively), and 86.4% of the cohort was ≥50 years of age (Table 1). Of the 29,570 ENT BSI-positive admissions evaluated, 25,479 (86.2%) patients received AET, 4,091 (13.8%) received IET, and 415 (1.4%) patients had carb-NS pathogens (Table 1). The average TAT for AST was 60.2 h (SD, 71.3). The observed mean post-BSI event LOS for carb-NS patients was almost twice that of carb-S patients (18.30 days; SD, 27.41 vs 9.63 days; SD, 10.16, respectively), whereas observed post-BSI event mortality was 18.5% (30 events) in the carb-NS group vs 6.4% (803 events) in the carb-S cohort (Table 1). Carb-NS infections were more likely to receive IET compared to carb-S infections (53.0% vs. 13.3%, respectively) (Table 2). Most healthcare facilities were in urban areas (83.2%), and over 50% of them had >100 beds (Table S2).

**TABLE 2:** Descriptive statistics of the *Enterobacterales* cohort^*a*^

ENT-blood	ENT cohort N (%)	IET events N (%)	AMR (carb-NS) events N (%)
Overall	29,570 (100)	4,091 (13.8)	415 (1.4)
Setting			
Admission	26,489 (89.6)	3,307 (12.5)	250 (0.9)
Hospital	3,081 (10.4)	784 (25.4)	165 (5.4)
AMR (carb-NS)			
S	29,155 (98.6)	3,871 (13.3)	0 (0.0)
NS	415 (1.4)	220 (53.0)	415 (100)
Empiric therapy (regrouped)			
IET	4,091 (13.8)	4,091 (100)	220 (5.4)
AET	25,479 (86.2)	0 (0.0)	195 (0.8)
Turnaround time (h)			
0-Q1 (1st Q, ≤42 h)	7,283 (24.6)	1,914 (26.3)	57 (0.8)
Q1-Q2 (median, 43–56 h)	7,470 (25.3)	712 (9.5)	49 (0.7)
Q2-Q3 (3rd Q, 57–65 h)	7,672 (25.9)	680 (8.9)	84 (1.1)
Q3 over (> 65 h)	7,145 (24.2)	785 (11.0)	225 (3.1)

^*a*^ ENT, *Enterobacterales*; AMR, antimicrobial resistance; S, susceptible; Carb-NS, carbapenem non-susceptible; IET, inadequate empiric therapy; AST, antimicrobial susceptibility testing; TAT, turnaround time; BSI, bloodstream infection; AET, adequate empiric therapy; LOS, length of stay; ICU, intensive care unit; Q, Quartile.

**Fig 1:**
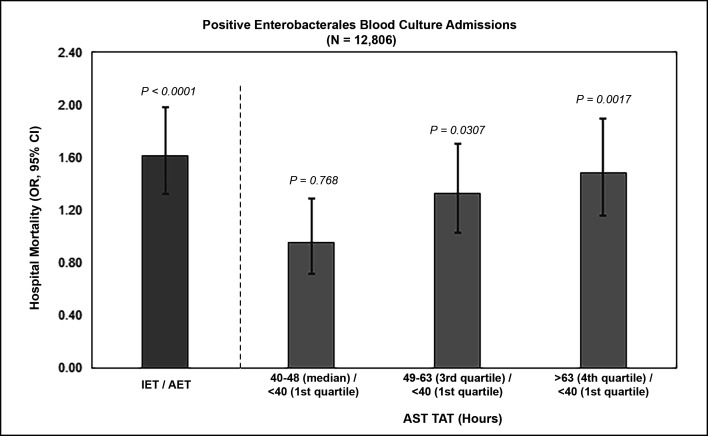
Model-estimated (adjusted) odds ratio of in-hospital mortality with positive *Enterobacterales* blood culture by AST TAT. Data presented here are based on available mortality data reported by facilities, and the quartile categories for TAT differ from those of the full population analysis.

The adjusted odds of post-BSI event mortality were 1.61 times higher for the IET group compared to the AET cohort [OR: 1.61 (95% CI, 1.32, 1.98); *P* < 0.0001] (Fig. 1). Longer AST TAT also correlated with higher odds of mortality, that is, AST TAT between 49 and 63 h had OR: 1.32 (95% CI, 1.03, 1.71; *P* = 0.0307), and AST TAT >63 h had OR: 1.48 (95% CI, 1.16, 1.90; *P* = 0.0017) compared to the reference (AST TAT <40 h) (Fig. 1).

**Fig 2:**
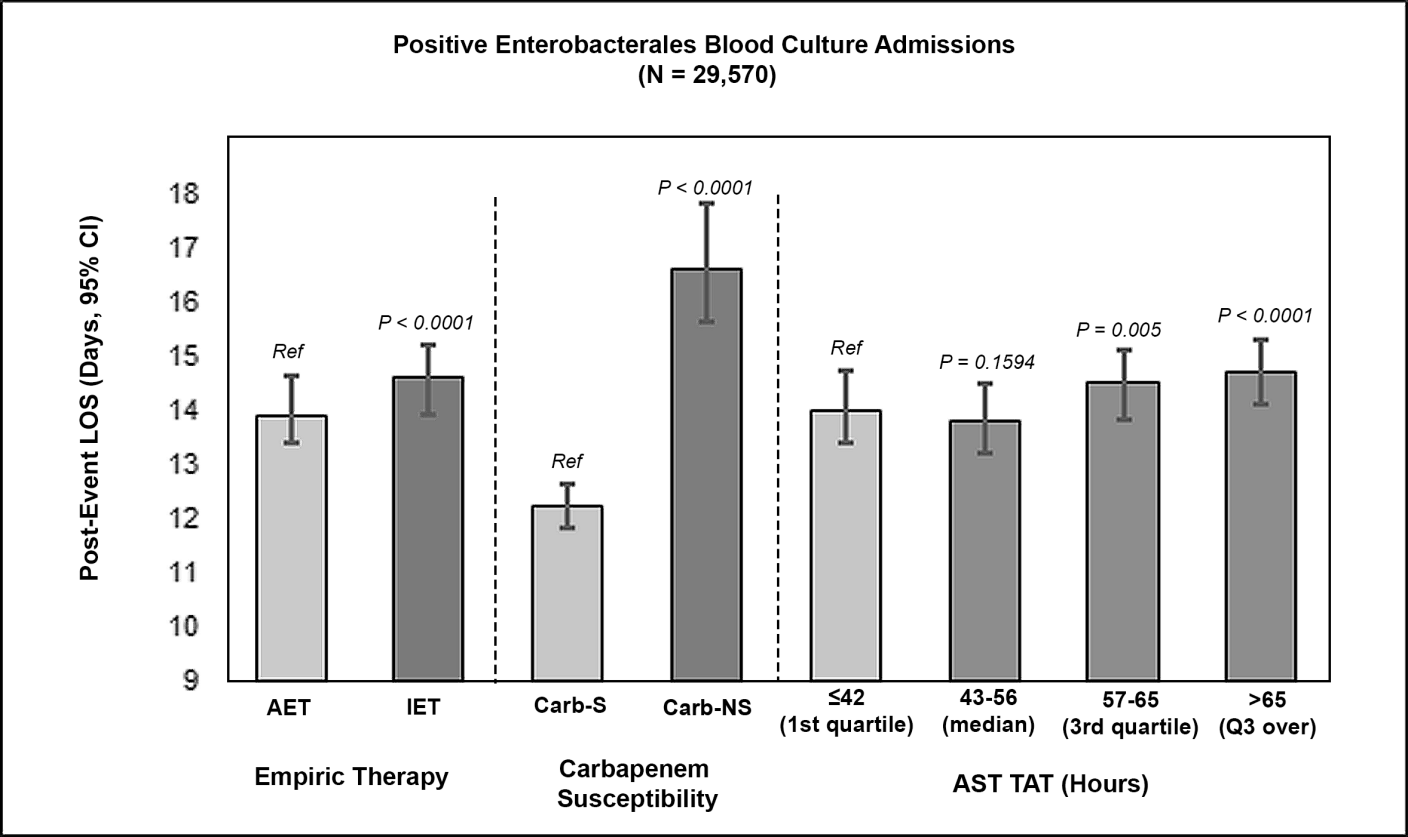
Model-estimated (adjusted) post-BSI event length of stay (days) with positive *Enterobacterales* blood cultures by empiric therapy, carbapenem susceptibility, and AST TAT. Results are adjusted for covariates. Post-BSI events LOS are presented with 95% CI.

In the adjusted analysis using multivariable regression modeling, the post-BSI event LOS for the IET group was slightly, albeit significantly longer than the LOS in the AET cohort [14.6 days (95% CI, 13.9, 15.2) vs 13.9 days (95% CI, 13.4, 14.6), respectively, *P* < 0.0001] (Fig. 2). Extended TAT was associated with longer LOS with AST TAT results >65 h from culture collection (*P* < 0.0001) (Fig. 2). Of the ENT-positive cohort, the carb-NS group had a significantly higher post-BSI event LOS compared to the carb-S cohort [16.6 days (95% CI: 15.6, 17.8) for carb-NS vs 12.2 days (95% CI: 11.8, 12.6) for carb-S; *P* < 0.0001] (Fig. 2).

## DISCUSSION

Our study demonstrated that inadequate empirical antibacterial therapy, delayed AST TAT, and ENT carb-NS pathogens are associated with adverse patient outcomes. While poor outcomes for patients with BSI often involve factors that are outside of the healthcare facilities sphere of influence (i.e., Pitt score >2, lung and abdomen as primary site of infection, Charlson score >4, and immunosuppression) (12), our study showed that certain variables within hospital control, such as time to result and downstream clinical antimicrobial optimization to adequate therapy, can significantly impact patient outcomes. BSI outcomes hold contemporary clinical relevance as reports from health departments in England and Germany have indicated disproportionally poor outcomes. Likewise, in the US, the mandatory central line bloodstream infection (CLABSI) and newly proposed Hospital Onset Bacteremia (HOB) metrics are both associated with higher mortality, length of stay, and cost (13–15). In this context, improving diagnostic stewardship workflows may have the potential to identify the offending pathogen and ostensibly administer the most appropriate antimicrobial sooner, therefore optimizing the chance of alleviating the deleterious outcomes of a BSI.

In our study, the post-BSI event mortality overall was 61% higher for patients started on inappropriate empiric therapy compared to appropriate empiric therapy, with longer AST TAT showing significant correlations with increasing post-BSI event mortality. Although less than 1 day, a significant increase in LOS for post-BSI IET was also observed when compared to post-BSI event LOS in the AET cohort. In a recent publication, hospital-onset bloodstream infections incurred an incremental cost of $22,000–$55,000, approximately three times the mortality risk, and an incremental 10–17 additional LOS days compared to matched controls (15). Our analysis builds upon those insights by focusing on all-comers (i.e., community-onset and hospital-onset period) and specifically for ENT BSI events. More specifically, by focusing on clinical outcomes based on specific phenotypes [*Enterobacterales* in aggregate are the predominate pathogens in hospital-acquired infections (16, 17)] and time differential from blood culture acquisition and AST results, we quantify the areas in workflow that microbiology laboratories and antimicrobial stewardship programs (ASPs) can realistically influence. In fact, in March 2020, the Centers for Medicare and Medicaid Services (CMS) codified in their ruling of ASPs that such programs are required and must be connected to infection prevention and quality executive hospital leadership (18), which is also reflected in The Joint Commission (TJC) hospital licensing surveys. In this new paradigm, in the US at least, there is now an infrastructure required by CMS and TJC to address the clinical care gaps presented in this study. The United Kingdom and Germany have similarly created quality metrics based on specific types of bloodstream infections that intersect with our study and reflect the quality issues related to this ubiquitous problem in healthcare (13, 14). By identifying incremental increases in post-BSI event LOS and mortality associated with IET, delayed time to AST results, and carb-NS status, there are operational workflow opportunities that may positively affect patient care. For example, most hospitals will provide an antibiogram which summarizes facility-level susceptibility to the most commonly occurring pathogens encountered in the aggregate patient case mix. By leveraging trends of resistance by pathogen type, clinicians can become acquainted with a reference predicated on their own patient population that can help guide better empirical therapy choices when a BSI is suspected. Hence, whereas antibiotic resistance and inappropriate prescribing of antibiotics are well-recognized issues among physicians, there is still little uniformity across healthcare facilities on the use of stewardship activities to better inform prescribing decisions. A recent national survey on antibiotic resistance and antibiotic stewardship in the US revealed that 82% of respondents would likely use reports on antibiotic resistance patterns pertaining to their geographic area to implement stewardship activities in their outpatient practice if these reports were provided by health departments (19). We, therefore, suggest that providing locally focused antibiograms on a routine cadence to physicians would provide them with actionable information upon which to base their respective stewardship activities. Regional variation in multi-drug resistance organisms (MDRO) and resistance over time has also been reported; therefore, maintenance of local antibiograms on a routine cadence can be an important asset to prescribing physicians.

For AST TAT, processes that decrease the time from blood culture collection to pathogen identification and subsequent time to accurate AST finalization may partially mitigate the associated higher LOS and mortality seen in this study. Adoption of best practices to optimize the reporting of blood culture results and minimize the risk of contamination and the need to repeat cultures is an example of such processes. Other examples include near-patient/decentralized blood culture incubation and Gram stain, couriers to expedite receipt by a centralized laboratory in multi-hospital healthcare systems, quality workflows to optimize pathogen isolation by meeting blood culture volume thresholds (20, 21), the use of automated blood culture systems and optimized blood culture media (22, 23), rapid clean-up products for direct identification (ID) from blood culture media (24, 25), matrix-assisted laser desorption/ionization-time of flight (MALDI-TOF) mass spectrometry for organism ID (26, 27), qualitative multiplex nucleic acid-based *in vitro* diagnostic systems (28), direct AST from positive blood culture platforms (29–31), and conventional automated ID/AST systems (32, 33). As antimicrobial stewardship programs are required by CMS and supported by TJC since March 2020, this analysis delineates the clinical care gap outcomes that can inform specific goals of antimicrobial stewardship teams by providing a benchmark derived from national data to juxtapose local clinical gains made through more timely interventions when targeting disease states such as ENT BSI. Finally, the findings in this study are a call to industry for innovation in diagnostics that detect and provide, in a timely manner, AST results for resistant pathogens associated with high mortality and longer LOS, such as CRE.

### Limitations

The observational nature of the study we conducted is one of its inherent limitations. In addition, microbiology results were those of each healthcare facility where patients were treated and as such, no remnants of specimens were available for the study team to perform confirmatory testing. Also, the database did not contain information on outpatient antibacterial exposure, and some of the geographic regions were underrepresented. The definitions of adequate and inadequate empirical therapy used in the investigation were also based solely on antimicrobial orders and, therefore, did not account for other variables that could impact medication effectiveness such as the dosage regimen administered. The impact of AST methods used by reference laboratories across facilities, the use of rapid diagnostics on AST TAT, and the duration of IET were not evaluated as part of this investigation. Although TAT to first AST results may influence findings, it is unlikely that rapid diagnostics would impact conventional time to AST results, which was the primary objective of our study. Furthermore, we did not evaluate rapid genotypic testing (or other rapid pathogen identification such as PCR, PNA-FiSH, for example) and their influence on conventional AST, if any. While typically genotypic and molecular testing is used in addition to traditional AST in a parallel workflow, it could be possible that a hospital instituted an internal “reflex” stepwise testing algorithm, which would be beyond the scope of this analysis. In addition, TAT can be influenced by bacterial burden, which we were not able to evaluate. Finally, the uniformity of testing methods, adherence to testing, and laboratory workflow processes across participating healthcare facilities could not be controlled. Given that CMS is currently reviewing Hospital Onset Bacteremia and Fungemia (HOB) (34) as a mandatory quality metric and that other countries such as England and Germany also have BSI quality metric initiatives, building on this momentum to address additional clinical care gaps with operationally viable solutions may prove most beneficial in improving the care of patients with BSI.

### Conclusion

In this retrospective multicenter analysis, we reported that IET, carb-NS, and delayed AST TAT are associated with adverse hospital outcomes when BSI is caused by ENT. Consequently, efforts should be made to accelerate accurate AST TAT for ENT isolated in blood to provide timely and adequate therapy to reduce hospital LOS and mortality. Moreover, given that IET is associated with worse outcomes, the integration of diagnostic and antimicrobial stewardship can improve the outcomes of patients with resistant ENT infections.

## Data Availability

Per contractual agreements, we are unable to share patient encounter-level data without restriction. The data sets/regression code used and/or analyzed during the current study are available from the corresponding author on reasonable request.

## SUPPLEMENTAL MATERIAL

Supplemental tables spectrum.00402-24-s0001.pdf Tables S1 and S2.

ASM does not own the copyrights to Supplemental Material that may be linked to, or accessed through, an article. The authors have granted ASM a non-exclusive, world-wide license to publish the Supplemental Material files. Please contact the corresponding author directly for reuse.
