# Supplementary material for: Hospital mortality and length of stay associated with Enterobacterales positive blood cultures: a multicenter analysis
Source: Microbiol Spectr. 2024 Jul 2;12(8):e00402-24. doi: 10.1128/spectrum.00402-24 (PMC11302144; doi:10.1128/spectrum.00402-24)
Supplement: Supplemental tables — Tables S1 and S2. [file spectrum.00402-24-s0001.pdf]

SUPPLEMENTAL MATERIAL

Supplemental Tables

Table S1. Laboratory criteria used to determine the presence of selected concurrent clinical conditions within five days of culture collection

Clinical Condition	Criteria
Lactic Acidosis	Serum lactate >4.0 mmol/L
Renal insufficiency/failure	Insufficiency: Serum creatinine > 2.0 mg/dL; Failure: Blood urea nitrogen >100 mg/dL AND serum creatinine >3.0 mg/dL
Heart failure	Brain natriuretic peptide (BNP) >400 pg/mL OR N-terminal BNP >900 pg/mL
Liver dysfunction	Any of the following: alanine aminotransferase >60 U/L, aspartate aminotransferase > 80 U/L, serum albumin <3.0 gm/dL, international normalized ratio >2.0 [and not currently receiving warfarin, rivaroxaban, apixaban, edoxaban, or betrixaban]
Cytokine storm	Any of the following: fibrinogen <250 mg/dL, C-reactive protein >70 mg/L, D-dimer >1000 ng/mL, erythrocyte sedimentation rate >30 mm/hr., triglycerides >265 mg/dL
Immunocompromised	Absolute neutrophil count <1,000 µL or absolute lymphocyte count <1,000 µL

Table S2. Hospital demographics distribution

**Category**	**Total (N=161)**
< 100 beds	66 (41.0)
100-300 beds	68 (42.2)
> 300 beds	27 (16.8)
East (North Central)	18 (11.2)
East (South Central)	38 (23.6)
Middle Atlantic	31 (19.3)
Mountain	2 (1.2)
New England	1 (0.6)
Pacific	15 (9.3)
South Atlantic	38 (23.6)
West (North Central)	2 (1.2)
West (South Central)	16 (9.9)
Rural	27 (16.8)
Urban	134 (83.2)
Non-teaching	101 (62.7)
Teaching	60 (37.3)
